# Photochemical Retinopathy induced by blue light emitted from a light-emitting diode Face Mask: A case report and literature review

**DOI:** 10.1097/MD.0000000000020568

**Published:** 2020-06-12

**Authors:** Tae Gi Kim, Junkyu Chung, Jisang Han, Kyung Hyun Jin, Jae-Ho Shin, Sang Woong Moon

**Affiliations:** a Department of Ophthalmology, Kyung Hee University Hospital at Gangdong; b Department of Medicine, Graduate School; c Department of Ophthalmology, Kyung Hee University Medical center, Kyung Hee University, Seoul, Korea.

**Keywords:** Blue light, light-emitting diode, light-emitting diode mask, photochemical retinal damage

## Abstract

**Rationale:**

Skin photobiomodulation involves the use of low doses of light of a specific wavelength to reduce skin inflammation and promote tissue repair. Recently, a face mask using a light-emitting diode (LED) to induce photobiomodulation has been widely introduced in the market. However, a short wavelength of high-energy blue light can cause retinal damage. We would like to report a rare case of photochemical retinopathy after using a blue LED face mask.

**Patient concerns:**

A 37-year-old woman presented with complaints of distorted vision. The patient was exposed to blue light from an LED face mask 1 month before presentation.

**Diagnosis:**

Color fundus photography revealed a yellowish chorioretinal lesion and optical coherence tomography revealed retinal pigment epithelium destruction in the parafoveal area. Fluorescein angiography revealed leakage from the lesion at the parafovea. The patient was diagnosed with blue LED-induced photochemical retinopathy.

**Interventions:**

Intravitreal bevacizumab was injected in the right eye.

**Outcomes:**

After 4 weeks, dysmorphopsia was improved.

**Lessons:**

This case report demonstrates that retinal damage can occur in humans due to prolonged exposure to blue light. Therefore, it is important to be wary of eye exposure and ensure the eyes are covered during LED face mask use.

## 1 Introduction

Altering cellular function using low-energy, non-thermal light-emitting diodes (LEDs) light is known as photobiomodulation.^[1]^ The depth of tissue penetration and the target site of treatment change depending on the wavelength of the LED light. In general, light in the spectral range from 600 to 1300 nm is useful for promoting wound healing, tissue repair, and skin rejuvenation.^[2–4]^ Recently, LED face masks for facial skin rejuvenation have been widely introduced to the market. Generally, the red LED emits light at a wavelength of 630 to 700 nm, while the infrared LED emits a wavelength of 800 to 1200 nm. Red and infrared light, which have relatively long wavelengths, penetrate deep into the tissue and facilitate the regeneration of dermal structures, such as adnexa and fibroblasts.^[5]^

Blue light has a wavelength of 400 to 470 nm and its tissue penetration depth is low because of its short wavelength. It has antibacterial and anti-inflammatory properties, and is mainly used for skin diseases such as acne.^[6–9]^ However, many animal studies have shown that intensive blue light can cause retinal cell damage and alter blood retinal barrier function.^[10–15]^ In mouse model, retinal damage was observed when exposed to blue LEDs for 2 hours.^[10,11]^ The LED-induced retina damage is known to be wavelength -dependent rather than energy-dependent.^[10]^ Therefore, the short wave length of the blue light LED itself can cause the retinal damage. However, to our knowledge, there have been no reports of retinal damage caused by long term exposure to blue light in the human eye.

We report a case of photochemical retinopathy after the long-term use of a blue LED face mask in a 37-year-old woman who was treated with intravitreal bevacizumab injection. No identifiable health information was included in this case report.

Informed written consent was obtained from the patient for publication of this case report and accompanying images. No ethical approval was obtained because this study is retrospective case report and did not involve a prospective evaluation.

## 2 Case report

A 37-year-old woman presented to the clinic with complaints of distorted vision in her right eye. The patient was exposed to blue light from an LED face mask (Cellreturn LED therapy mask, BUJA CO, Korea) for 20 min once every 2 days for skin care from 1 month before presentation. When using the LED face mask, the patient reported that she had opened her eyes and continued with the routine tasks of daily life, as recommended by the manufacturer. The patient's medical history was significant for laser in-situ keratomileusis (LASIK) surgery on her right eye 10 years previously; however, there was no other history of ocular or systemic disease, or contact lens wear.

**Figure 1:**
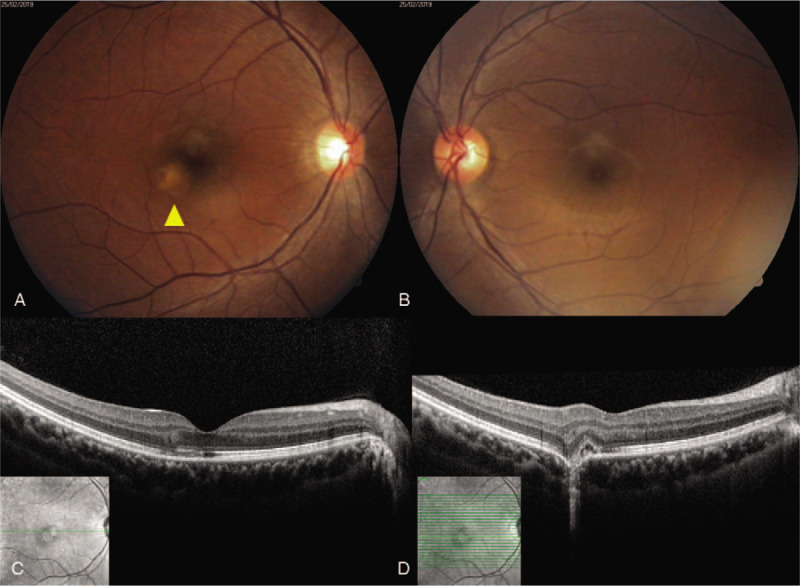
Fundus photography and optical coherence tomography (OCT) examination at the patient's first visit. (A) The right eye exhibited a yellowish chorioretinal lesion, noted in the parafoveal area. (B) No abnormalities are evident in the left eye. (C) OCT of the right eye revealed focal photoreceptor destruction at the fovea. (D) OCT of the right eye revealed subretinal fluid, destruction of photoreceptors, and retinal pigment epithelium. OCT = optical coherence tomography.

**Figure 2:**
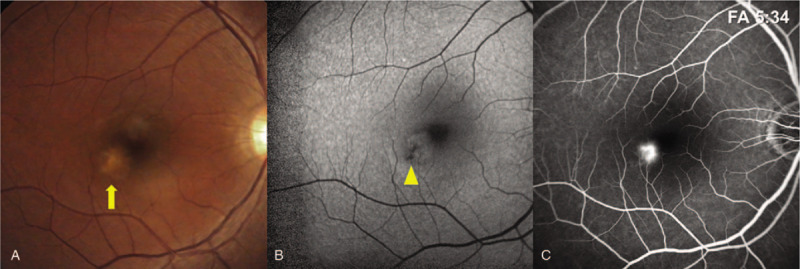
(A) Color fundus photography revealing a yellowish placoid lesion at the parafoveal area (arrow). (B) Fundus autofluorescence of the right eye revealing a circular area of hypo-autofluorescence compatible with retinal pigment epithelium destruction (arrow head). (C) Early fluorescein angiography revealing obvious dye leakage from the lesion at the parafovea.

**Figure 3:**
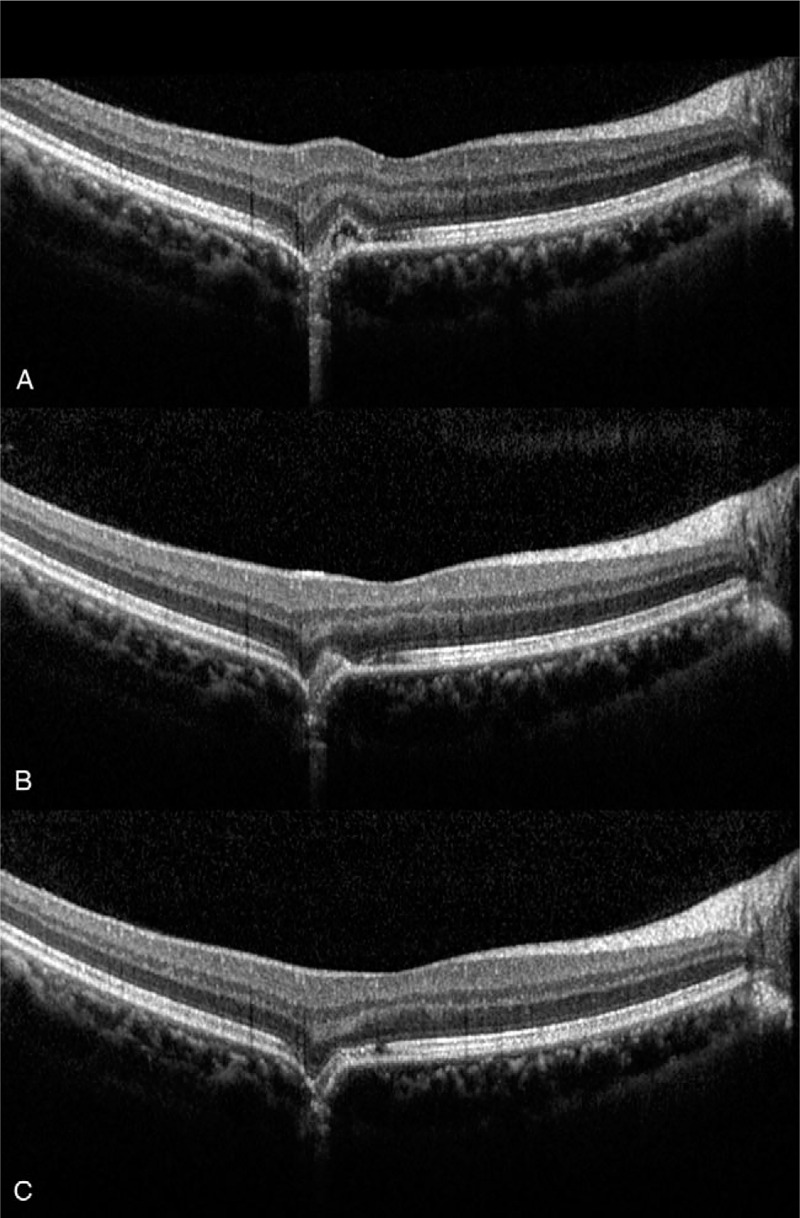
(A) Optical coherence tomography (OCT) at the time of presentation revealing subretinal fluid and destruction of the retinal pigment epithelium and photoreceptor layer. (B) One week after intravitreal bevacizumab injection, the subfoveal fluid has decreased. (C) Four wk after intravitreal bevacizumab injection, OCT revealed marked improvement of the photoreceptor layer; however, excavated choroidal contour persisted. OCT = optical coherence tomography.

On examination, the best corrected distance visual acuity was 20/20 in both eyes. Her intraocular pressure, measured using a non-contact tonometer, was 13 mm Hg in the right eye and 12 mm Hg in the left. The axial length was 24.4 mm in the right eye and 23.3 mm in the left eye. Automated refraction revealed sphere -0.25, cylinder -0.50, axis of 152 in the right eye, and sphere +0.25 in the left eye. Color fundus photography revealed a yellowish chorioretinal lesion without drusen (Fig. 1A). Optical coherence tomography (OCT) revealed retinal pigment epithelium destruction in the parafoveal area, with mild disruption of the photoreceptors in the foveal region. (Fig. 1C, D). Fluorescein angiography revealed leakage from the lesion at the parafovea (Fig. 2B). In autofluorescence imaging, hypo-autofluorescence due to retinal pigment epithelium destruction was observed (Fig. 2C). The patient was diagnosed with blue LED-induced photochemical retinopathy and intravitreal bevacizumab was injected. Seven days after injection, subretinal fluid decreased and best corrected distance visual acuity was 20/20 in the right eye. (Fig. 3B). After 4 weeks, dysmorphopsia symptoms were improved and the photoreceptor layer was improved on OCT; however, the site of disruption in the retinal pigment epithelium layer remained excavated (Fig. 3C).

## 3 Discussion

LED light exerts its photobiomodulation effects through mechanisms including increases in adenosine triphosphate concentration, modulation of reactive oxygen species, collagen synthesis alteration, angiogenesis, increased cellular proliferation, elevation of reactive oxygen species levels, and increased blood flow.^[16–19]^ Important cell types for photobiomodulation include fibroblasts, keratinocytes, and immune cells. Specific wavelengths of LED light penetrate tissues to varying depths to increase intradermal collagen density and reduce the signs of aging.^[20]^

**Figure 4:**
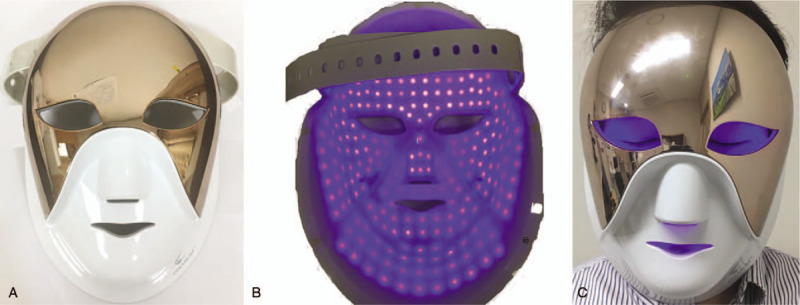
Light-emitting diode (LED) face mask in operation. (A) External surface of the LED face mask. The eye area is open without a protective cover. (B) The internal surface of the LED face mask during blue LED operation. Appearance and operation of the LED. (C) The area around the eyeball is exposed to blue light when wearing the LED face mask. LED = light-emitting diode.

In the present case, the LED face mask had a first light source that emitted a wavelength of 460 nm to 470 nm (blue light), a second that outputted a wavelength of 620 nm to 680 nm (red light), and a third that outputted a wavelength of 760 nm to 900 nm (infrared light). The mask is designed to wrap around the face, and the area around the eyes is opened without a cover so as not interfere with the activities of daily life (Fig. 4). According to the product manual, its recommended use is 5 to 20 minutes per day, >3 times per week, depending on the condition of the skin. The power of the blue LED light in this product, however, is not disclosed by manufacturer.

Blue light (wavelength range, 400 nm to 470 nm) has a subcutaneous penetration depth of approximately 1 mm. Blue light appears to exert its effect on acne via its influence on *Propionibacterium acnes* and anti-inflammatory properties.^[21]^ Blue light has antibacterial effects via the formation of oxygen free radicals, and the anti-inflammatory effect appears to be the result of a shift in cytokine production.^[22]^

However, short-wavelength blue light is associated with eye damage, especially retinal damage. If this high-energy blue light enters the retina through the cornea, it can cause diseases including age-related macular degeneration. Several previous animal studies have reported that blue light can lead to eye problems, particularly retinal damage and blood retinal barrier function abnormalities.^[10–15]^ According to the amplitude of electroretinogram, Kim et al^[10]^ reported that retinal degeneration was observed in mice after 2 h of blue LED (460 nm) irradiation. This wavelength is the same as the blue light wavelength used in the LED mask in this case. in addition, Nakamura et al^[11]^ reported that retinal degeneration was observed in the inner and outer segments of photoreceptor cells after blue light (456 nm) irradiation at 400 lx or 800 lx for 2 hour. Geiger et al^[12]^ reported that the destruction of the blood-retina barrier and retinal edema occurred when mice were exposed to blue light (410 ± 10 nm) at a power of 60 mW/cm^2^ for 10 to 30 min. Koide et al^[13]^ reported the threshold level for blue LED light (460 nm) was a 3 mm beam at 0.85 mW for approximately 40 minutes. The authors also reported that blue LED light caused a marked disruption of the disks of photoreceptor cells, damaged retinal pigment epithelium apical villi, and a loss of retinal pigment epithelium melanin after 90  minutes exposure. Other studies have reported that albino rat retinas exhibited apoptotic cell death when exposed to blue light (403 nm) but not when exposed to green light (550 nm).^[14]^

In human, Liang et al^[23]^ reported a case of macular damage caused by blue LEDs used for bar illumination. However, the exact blue light wavelength or power is not known and it is difficult to assume that macular damage is caused by blue light in the presence of multiple lights such as bars. In this case, it is meaningful to confirm that the retinal damage was caused by regular blue LED exposure.

Retinal damage in the patient in our case was presumed to be caused by direct photoreceptor and retinal pigment epithelium damage due to exposure of the retina to blue light above threshold levels for a long period of time. The reason for damage to the parafovea, not the fovea, is presumably because the blue light entered the retina obliquely and not exactly along the visual axis. Therefore, retinal cell damage in the parafovea is believed to be caused by direct exposure to blue light. This is supported by focal photoreceptor damage and dye leakage observed in retinal pigment epithelium destruction and fluorescein angiography in patients undergoing OCT.

This case should be distinguished from “focal choroidal excavation” known as congenital disease. Focal choroidal excavation is an unusual concavity in the choroid of unknown etiology occurring without posterior staphyloma or scleral ectasia and is known to cause central serous chorioretinopathy and choroidal neovascularization.^[24,25]^ In OCT, focal choroidal excavation showed preservation of the photoreceptor layer and RPE within the lesion. However, in this case, the photoreceptor layer and RPE in the lesion are destroyed (Fig. 1D). Therefore, the choroidal excavation seen in this case was presumed to be most likely caused by direct exposure to blue light.

## 4 Conclusion

In conclusion, retinal damage can occur in humans, as in this case, due to prolonged exposure to blue light. To our knowledge, the present case report is the first to describe photochemical retinal damage caused by long term use of blue LED in humans. Skin photobiomodulation using blue LED may be a proven method; however, given the risk for retinal damage, it is better to use red or infrared light with longer wavelength for retinal safety. Moreover, when using a blue LED facemask, it is important to be wary of eye exposure and ensure the eyes are covered during use.

## Author contributions

**Conceptualization:** Sang Woong Moon.

**Data curation:** Junkyu Chung, Jisang Han.

**Formal analysis:** Tae Gi Kim, Jae-Ho Shin, Kyung Hyun Jin.

**Funding acquisition:** Sang Woong Moon.

**Investigation:** Tae Gi Kim.

**Methodology:** Tae Gi Kim, Sang Woong Moon.

**Writing – original draft:** Tae Gi Kim, Sang Woong Moon.

**Writing – review & editing:** Tae Gi Kim, Sang Woong Moon.
